# The influence of disaster knowledge, official trust, and sources of warning information on public risk perception in typhoon-prone areas of China: a structural equation modeling analysis

**DOI:** 10.3389/fpubh.2026.1728646

**Published:** 2026-01-28

**Authors:** Ping Wei, Na Zhang, Fang Bai, Zhenyu Zhao, Yajuan Zhao, Xuren Wang, Zhongxia Wang, Chunhua Dai, Yani Lu, Siyuan Qin, Baichao Xu, Yuan Mei, Hua Zhang

**Affiliations:** 1 School of Nursing, Hainan Medical University, Haikou, Hainan, China; 2 Key Laboratory of Emergency and Trauma of Ministry of Education, The First Affiliated Hospital of Hainan Medical University, Haikou, Hainan, China; 3 Nursing Department, The First Affiliated Hospital of Hainan Medical University, Haikou, Hainan, China; 4 Nursing Department, Changhai Hospital, Naval Medical University, Shanghai, China; 5 Nursing Department, Hainan Health Vocational College, Haikou, Hainan, China; 6 Hainan Key Laboratory of Philosophy and Social Sciences for Sports and Health Promotion, Hainan Medical University, Haikou, Hainan, China

**Keywords:** disaster knowledge, disasters, official trust, risk perception, sources of warning information, structural equation modeling, typhoon

## Abstract

**Background:**

Typhoons are one of the most common natural disasters, seriously jeopardizing public safety. Risk perception, defined as the subjective judgment that people make about the characteristics and severity of a risk, plays a crucial role in public preparedness behavior during typhoon disasters. However, there is a lack of knowledge regarding public risk perception and its influencing factors in typhoon-prone areas. This study aims to fill the knowledge gap by evaluating the public risk perception and key factors associated with perceptions among the general public. In addition, the relationship between the disaster knowledge, official trust, sources of warning information, and risk perception will be explored.

**Methods:**

A cross-sectional study was conducted from October 2024 to January 2025 with participants from Hainan Island, China. The target population consisted of residents aged 18 years and older who had resided on Hainan Island for over half a year (*N* = 517). The perceptions related to typhoon disaster were collected with the Typhoon Disaster Public Risk Perception Scale (TDPRPS). Ordered Choice Model theory was used to develop a hypothesized model to test hypotheses regarding residents’ disaster risk perception, and structural equation modeling (SEM) was used to test the model.

**Results:**

Out of 517 participants surveyed, Data were comprised of 517 valid responses from 11 major administration areas across Hainan Island. Disaster knowledge, official trust, sources of warning information were significantly correlated with public risk perception. SEM results revealed that the model fit the data well. The results showed that disaster knowledge and official trust are directly and positively associated with risk perception (the unstandardized coefficients of direct path are 0.331 and 0.467 respectively, *p* < 0.01). While the relationship between source of warning information and risk perception was fully mediated by disaster knowledge and official trust (the unstandardized coefficient of indirect path is 0.211, *p* < 0.001).

**Conclusion:**

This study indicates that warning information does not directly correlate with risk perception; their relationship is fully mediated by disaster knowledge and official trust. To improve disaster risk perception, government agencies might consider strengthening institutional credibility, diversifying warning channels, and enhancing public disaster education.

## 1 Introduction

The World Health Organization (WHO) reports that global climate change and natural disasters—including earthquakes, tropical cyclones, floods, landslides, wildfires, and droughts—are increasing in both frequency and intensity, posing serious threats to public health safety (1). Tropical cyclones are among the most common natural disasters worldwide (2). These cyclones, known as typhoons, are extremely destructive and often accompanied by strong winds, heavy rain, and floods. The impact of typhoon disasters in China is profound (3). The National Meteorological Disaster Risk Census report (2024) documents that, from 1989 to 2021, these disasters led to 11,919 deaths and missing persons, as well as direct economic losses of 180 billion USD (4). Hainan Island, China, is recognized as a “typhoon corridor.” Typhoons and related disasters there result in more casualties than any other natural hazard (5). On September 6, 2024, Super Typhoon Yagi made landfall on Hainan with sustained winds over 200 km/h. It was one of the strongest autumn typhoons in China since 1949 (6). The disaster affected about 7.415 million people, with direct economic losses preliminarily estimated at 9.9 billion USD (7).

The public acts as both victims and key actors in typhoon disaster mitigation. Thus, the final impact of a disaster is shaped by the interactions of human activities within the disaster scenario. Research suggests that a psychological perspective better explains the interaction effects of human precautionary behavior (8). Prior research has already established the correlation between disaster preparedness and various psychological factors, such as risk perception, behavioral intention, and self-efficacy (9–11). Among these, risk perception is a key process for understanding resident behavioral response to natural disasters (8, 9). Risk perception is an intuitive risk judgment by the public when assessing the threat of disasters, further leading to the process of decision-making (12). While the link between risk perception and protective behavior is established (13–15), a critical practical challenge persists: why do individuals often perceive and respond to the same warning information differently? Simply analyzing risk perception level is insufficient. Effective risk communication and policy design require a deeper understanding of how this perception is formed. Specifically, the mechanisms through which different sources of warning information are filtered and interpreted by individuals’ disaster knowledge and their official trust remain inadequately explored. Addressing this gap is essential to moving from generic warnings to targeted communication strategies that can effectively elevate public risk perception and prompt action.

The Ordered Choice Model (OCM) theory states that human decision making about warnings resembles an ordered-choice decision process. It was first proposed by American natural disaster expert Mileti in 1990, consists of three stages: hear, process, response (16). The process is initiated by hearing an initial warning. Therefore, the source of warning information can be regarded as a critical factor during the hearing stage. Subsequently, in process stage, people go through a gradual process in which they consider various decision factors before taking action, which leads to various psychological and behavioral outcomes. The OCM theory summarizes this process into three aspects: understanding, believing, personalizing. Studies showed that disaster knowledge can effectively help understand early warning information (17). And in risk communication, official trust can also enhance the response to warning information (18, 19). Warnings are more accurately personalized if they are understood and believed, leading individuals to perceive the threat (i.e., risk perception) (11). Meanwhile, receiver factors also influence the entire process. Thus, OCM theory provides an effective theoretical framework for the processing of warning information in various disasters and is widely used in risk communication and preparedness decision-making (20, 21).

The dissemination of warning information is crucial to the public’s risk perception. Both the source and the content of the information significantly influence the public’s risk perception of the event (22). With the advancement of information technology, there are various sources of information, which typically include the government, official news media, social media, and individuals in their vicinity (23, 24). Most people would balance their evacuation options based on warning information of typhoons obtained through different sources, taking into account the safety of their family and property (25, 26). The preference for information sources can significantly affect their level of disaster risk perception. Kasperson indicated that disasters interact with cultural, institutional, social, and psychological processes in ways that may amplify or attenuate public responses to the risk or risk event (27). While previous studies have established an association between source of warning information and risk perception, their underlying mechanism remains unclear. Further research is necessary.

Previous studies have suggested that the public’s level of risk perception is related to disaster knowledge and influences individual disaster response behaviors (28). Disaster knowledge is essential for effective preparedness before disaster. It serves as the theoretical premise that enables residents to take adequate protective measures (17). Generally speaking, disaster knowledge of typhoon mainly includes disaster consequences, disaster risk judgment and disaster response measures (11, 17, 29). It is worth noting that previous studies have found contradictory results regarding the impact of disaster knowledge on risk perception (10). A post-earthquake study found a positive association between disaster knowledge and both risk perception and preparedness (17). In contrast, Thomas reported no significant correlation between disaster knowledge and either risk perception or preparedness when a disaster is imminent (30). However, the relationship between individual disaster knowledge and risk perception specifically in the context of receiving early typhoon warnings remains unclear.

Official trust plays an essential role in disaster management. Studies show that official trust enables people to interpret warning information appropriately, and perceives risk that encourages the adoption of protective behaviors (31). Official trust is fundamental for the successful implementation of disaster risk communication strategies (19). When residents cannot perceive the risks that are coming, then public decisions are guided by official trust (32). Previous studies have shown that trust factor and risk perception are in inverse relationship (33). When facing the risks of natural hazards, this entails high uncertainty. Official trust thereby serves to mitigate this uncertainty (34). Based on this, high official trust may reduce risk perception. Before the typhoon comes, whether residents believe the warning information may depend on the official trust. However, there is no consensus on the role of official trust in the public risk perception of typhoon disasters.

**Figure 1:**
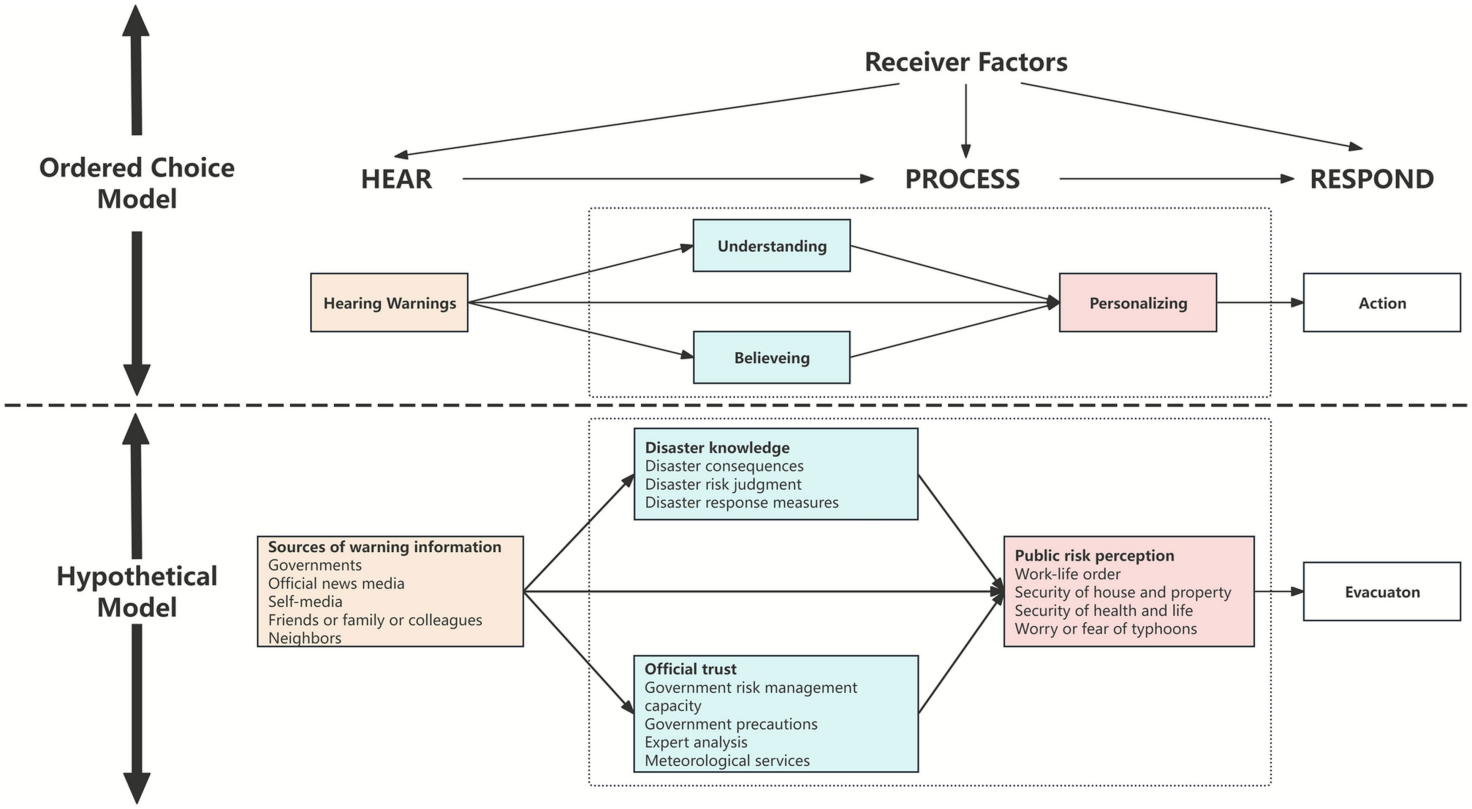
Hypothetical model of this study.

Every disaster situation presents new challenges, both for authorities and the public handling the typhoon disaster. This is especially true for the general public—the primary agents in typhoon disaster response. Their proactive preparedness and risk-avoidance behaviors can significantly reduce disaster-related losses. Therefore, understanding public risk perception is crucial for guiding their disaster responses, such as evacuation behavior. However, empirical studies on public risk perception in the immediate aftermath of typhoon disasters remain scarce. These studies often lack theoretical frameworks and fail to clarify the relationships between key variables. This study aims to address this knowledge gap. Specifically, there is insufficient research on how individuals, after receiving warning information, process disaster knowledge and official trust to ultimately internalize their risk perception. The OCM theory provides an effective theoretical framework and outlines three stages of hear, process (which includes understanding, believing, personalizing), and response. Based on this, a hypothetical path model of this study is presented in Figure 1. The specific hypotheses are as follows:

*Hypothesis 1 (H1):* Public risk perception can be directly associated with disaster knowledge.

*Hypothesis 2 (H2)*: Public risk perception can be directly associated with official trust.

*Hypothesis 3 (H3)*: Public risk perception can be directly associated with source of warning information.

*Hypothesis 4 (H4)*: The disaster knowledge and official trust mediates the relationship between risk perception and sources of warning information.

## 2 Materials and methods

### 2.1 Study design

The cross-sectional design was used in this study to enable a comprehensive analysis of the public’s risk perception across various demographic groups and to identify potential influencing factors (35). By concentrating on a representative population, this study aimed to draw generalized conclusions that could inform public health strategies and typhoon disaster prevention interventions tailored to those impacted by natural disasters. This research has been registered on the Medical Research Registration and Filing Information System of China (HSR-25-000514).

### 2.2 Setting

The data were collected in Hainan Island, China. Located in a coastal area and at low altitude in the southernmost part of China, Hainan Island lies within the typhoon zone of the West Pacific and the South China Sea. Surrounded by the sea, it is frequently affected by typhoons each year. With a total of 10.48 million inhabitants, Hainan forms a high-density exposure zone where above 90% of settlements are within typhoon floodplains (36). From 1949 to 2020, 531 typhoons influenced Hainan, with 142 making landfall on the island (5). On average, 7.4 typhoons impact Hainan annually, directly exposing about 9.3 million residents to recurrent disaster risk.

### 2.3 Participants

Participants were members of the Hainan community. They were eligible to be included in this study as follows: (1) Participants lived in Hainan for more than half a year. This criterion was essential to focus on the short-term impact of typhoons on the public risk perception. (2) They were able to understand the content of the survey. Comprehension-check questions were incorporated into the questionnaire to screen respondents by their ability to comprehend key survey items. (3) Age ≥ 18 years old, and (4) consented and volunteered to participate in the study.

Participants were excluded from this study if they (1) had a history of psychiatric disorders or were currently using psychiatric medication, or (2) could not understand the Chinese language.

### 2.4 Sample size

The sample size in this study was calculated based on the widely accepted rule of thumb in structural equation modeling (SEM) that recommends 10–20 times the observed variables or items (37). Given the 16-item final instrument, a sample size of 160 to 320 was required to satisfy the sample-size-to-parameter ratio for the SEM model. We targeted 400 participants to accommodate potential exclusions due to non-response or exclusion criteria.

### 2.5 Recruitment

Participants were recruited using a convenience sampling method. It is suitable for our study because the time-sensitive nature of post-typhoon risk perception assessment prioritizes rapid enrollment. Possible participants were reached in different channels to ensure a more comprehensive study sample: (1) In Social Media (WeChat, Red Note, and TikTok), which are three most popular social media platforms in China; (2) Local Red Cross; and (3) University Networks: emails were distributed to all faculty, staff, and students via the Campus Public Platform.

### 2.6 Instrument

Demographic Questionnaire: The demographic questionnaire used in the study was developed by reviewing previous literature (14, 38, 39). The demographic information related to gender, age, educational attainment, household size, live with older adult(s) or have children, number of children, personal monthly income, residential status (local/non-local), length of residence, housing type, housing age, and disaster experience among respondents was collected.

Typhoon Disaster Public Risk Perception Scale (TDPRPS): The TDPRPS was developed by Dr. Sun, an expert on meteorological disasters in China, for localized assessment of public risk perception regarding typhoon disasters, published in 2018 (40). The development of this scale contextualized China’s typhoon disaster patterns and systematically integrated four validated theoretical dimensions from prior studies: (1) The official trust dimension synthesizing Renn’s five trust attributes and Kasperson’s social commitment framework (41, 42). (2) Disaster knowledge adapted from Powell’s risk communication and perception theory (43). (3) The warning information dimension refers to the research of Burnside (44). (4) The risk perception dimension derived from Peacock’s hurricane risk perception scale (13). The TDPRPS comprises 16 items rated on a 5-point Likert scale (1 = completely disagree, 5 = strongly agree). In the present study, the internal consistency (Cronbach’s *α*) for each subscale and the total scale is presented in Table 1. In its original development, the scale demonstrated good reliability, with subscale α coefficients ranging from 0.802 to 0.936 and an overall α of 0.795 (40). It has been widely used in research on major disaster events (45). Formal permission to use the scale was obtained via email from the original authors.

**Table 1:** Dimensions, items, and internal consistency of the TDPRPS.

Dimension	Item	Number of items	Total Score	Cronbach’s *α* (this study)
Sources of warning information	Governments; Official news media; Self-media; Friends or family or colleagues; Neighbors;	5	25	0.909
Disaster knowledge	Disaster consequences; Disaster risk judgment; Disaster response measures;	3	15	0.786
Official trust	Government risk management capacity; Government precautions; Expert analysis; Meteorological services;	4	20	0.962
Risk perception	Work-life order; Security of house and property; Security of health and life; Worry or fear of typhoons	4	20	0.749
Total scale	–—	16	80	0.852

All items were rated on a 5-point Likert scale.

### 2.7 Procedure

The data collection from participants began in October 2024 and concluded in January 2025. Data collection was conducted online using the “Wenjuanxing” platform^1^, a secure and professional online questionnaire service in China. The survey was accessible via multiple device types, including smartphones and computer web-browsers. Access to the survey was generated through the link or QR code. Individuals first read an electronic information letter outlining the study’s purpose, the right to voluntary participation, and privacy protections. After electronic informed consent, the participant continued to respond to survey items. Filling out the survey took about 10 min to complete. All items were set as mandatory.

### 2.8 Statistical analysis

Before analysis, the data were double-checked. The P–P plots showed that the data demonstrated a roughly normally distributed, and the absolute values of skewness and kurtosis for all observed variables were within acceptable limits (|skewness| < 2, |kurtosis| < 7). Continuous variables conforming to normal distribution were described using mean ± standard deviation (SD), and the t-tests and analysis of variance (ANOVA) were used to assess group differences. Categorical variables were expressed as numbers and percentages. Pearson correlation analysis was used to evaluate the correlations between disaster knowledge, official trust, sources of warning information and risk perception. A structural equation model (SEM) was specified and estimated using maximum likelihood to investigate the path relationships and to test the hypothesized mediation effects. The model’s goodness of fit was evaluated by using the following indicators: The chi-square to degrees of freedom ratio (*χ^2^/df* < 5.000), the Root Mean Square Error of Approximation (RMSEA < 0.080), the Goodness of Fit Index (GFI > 0.900), the Comparative Fit Index (CFI > 0.900), the Incremental Fit Index (IFI > 0.900), Tucker–Lewis Index (TLI > 0.900). When all conditions were satisfied, the SEM demonstrated a better fit and was strongly supported by the observed data (37, 46, 47). In addition, the bias-corrected bootstrapping method was employed, which is widely valued within the SEM framework for enhancing analytical power and controlling Type I errors (11, 48, 49). Therefore, based on the results of path analysis, the indirect effects of sources of warning information and risk perception (that is, the mediations via the constructs of the theory of OCM) were assessed using 95% confidence intervals from 2000 bootstrap samples. Indirect effects were regarded as statistically significant if the 95% confidence interval (95% CI) for the mediating pathway did not include 0. The threshold for statistical significance is *p* < 0.05 (two-tailed). The data were analyzed using AMOS 26.0 and IBM SPSS Statistics 27.0.

### 2.9 Ethical considerations

The study was assessed by the Ethics Committee of Hainan Medical University (Approval Number: HYLL-2024-784). The study procedures followed the principles of the Declaration of Helsinki (50). All participants joined the study based on their free will, and their informed consent was guaranteed. All received written information about the study, such as the introduction and the purpose of the study, procedures involved, potential risks and benefits, rights of participants, and assurances regarding confidentiality and voluntary participation. It also clearly informed the potential participants that their involvement was completely voluntary, and they could withdraw from the research at any time without any negative impact.

In addition, three measures were implemented to guarantee data confidentiality. Firstly, data collection was conducted through online, anonymous surveys to ensure that participant information could not be traced. Secondly, digital data was securely stored and encrypted for protection, accessible solely to study members. Third, all study members received ethics training, including participant privacy, data protection, and correct data handling.

## 3 Results

### 3.1 Participants’ demographic characteristics and associations with the risk perception

A total of 581 questionnaires were distributed, and 517 filled forms were included in the analysis (response rate: 88.98%); 64 invalid questionnaires were excluded due to uniform responses across all items, contradictory answers, and completion time within 3 min. The survey encompassed 11 major administration areas across Hainan Island. Of the 517 participants, 222 (42.94%) were male and 295 (57.06%) were female. The majority were aged 20–39 years (347, 67.12%), held a bachelor’s degree (403, 77.95%), and were local residents (397, 76.79%). A significant proportion had lived in Hainan for over 5 years (445, 86.07%) and had prior disaster experience (471, 91.10%). Regarding household composition, 230 participants (44.49%) reported a household size of 2–4 persons, 265 (51.26%) lived with older adult(s), and 266 (51.45%) had children, of whom 143 (27.66%) had more than two children. Economically, 266 participants (51.45%) reported a monthly personal income of 5,000 CNY or more. In terms of housing, 230 (44.49%) resided in commercial housing, and 236 (45.65%) lived in buildings aged 10–30 years. Moreover, univariate analysis revealed that the length of residence, gender, age, educational attainment, household size, have children, number of children, and disaster experience were statistically significant, *p* < 0.05. Demographic characteristics and associations with risk perception are presented in Table 2.

**Table 2:** Participants’ demographic characteristics and the scores of the risk perception (*N* = 517).

Characteristic	Category	*N*(%)	Risk perception		
			Mean ± SD	*t*/*t*’/*F*	*p*
Residential status	No	120(23.21)	15.83 ± 1.92	−1.869	0.063
	Yes	397(76.79)	16.23 ± 2.36		
Length of residence (years)	<5	72(13.93)	15.57 ± 1.89	−2.299	**0.022**
	≥5	445(86.07)	16.23 ± 2.31		
Gender	Male	222(42.94)	15.00 ± 2.03	−10.974	**<0.001**
	Female	295(57.06)	16.99 ± 2.05		
Age (years)	18–20	38(7.35)	15.18 ± 2.08	5.913	**0.001**
	20–39	347(67.12)	16.19 ± 2.26		
	40–59	119(23.02)	16.47 ± 2.2		
	≥60	13(2.51)	14.38 ± 2.43		
Educational attainment	High school and below	59(11.41)	15.53 ± 2.29	3.183	**0.042**
	Bachelor	403(77.95)	16.17 ± 2.30		
	Master and above	55(10.64)	16.56 ± 1.87		
Household size (Persons)	1	110(21.28)	15.27 ± 2.34	13.207	**<0.001**
	2–4	230(44.49)	16.15 ± 2.26		
	≥5	177(34.23)	16.66 ± 2.08		
Live with older adult(s)	No	252(48.74)	16.03 ± 2.33	−1.071	0.285
	Yes	265(51.26)	16.24 ± 2.21		
Have children	No	251(48.55)	15.48 ± 2.24	−6.631	**<0.001**
	Yes	266(51.45)	16.76 ± 2.12		
Number of children	0	251(48.55)	15.48 ± 2.24	22.012	**<0.001**
	1	123(23.79)	16.75 ± 2.19		
	≥2	143(27.66)	16.76 ± 2.07		
Personal monthly income (CNY)	<5,000	251(48.55)	15.94 ± 2.42	−1.955	0.051
	≥5,000	266(51.45)	16.33 ± 2.10		
Housing type	Self-built house	75(14.50)	16.12 ± 2.60	2.104	0.123
	Commercial housing	230(44.49)	16.35 ± 2.16		
	Rental house/dormitory	212(41.01)	15.91 ± 2.25		
Housing age (years)	<10	249(48.16)	16.12 ± 2.34	0.028	0.972
	10–30	236(45.65)	16.14 ± 2.18		
	≥30	32(6.19)	16.22 ± 2.46		
Disaster experience	No	46(8.90)	15.5 ± 1.67	−2.608	**0.011**
	Yes	471(91.10)	16.2 ± 2.31		

Bold *p*-values indicate statistical significance at *p* < 0.05.

### 3.2 Correlation between variables

Table 3 presents the mean scores, standard deviation and correlations of the variables of interest. The results of the Pearson correlation analysis indicated that public risk perception of typhoon disasters was positively correlated with disaster knowledge (*r* = 0.344, *p* < 0.01), official trust (*r* = 0.390, *p* < 0.01), and the source of warning information (*r* = 0.315, *p* < 0.01). Disaster knowledge was positively correlated with official trust (*r* = 0.391, *p* < 0.01) and source of warning information (*r* = 0.390, *p* < 0.01). Additionally, official trust was positively correlated with the source of warning information (*r* = 0.413, *p* < 0.01).

**Table 3:** Means, standard deviations, and correlation between disaster knowledge, official trust, sources of warning information and risk perception (*N* = 517).

Variables	Mean ± SD	1	2	3	4
1. Risk perception	16.14 ± 2.27	1			
2. Disaster knowledge	12.54 ± 1.77	0.344**	1		
3. Official trust	16.82 ± 1.84	0.390**	0.391**	1	
4. Sources of warning information	20.26 ± 4.69	0.315**	0.350**	0.413**	1

**P < 0.01.

### 3.3 Structural equation modeling results

**Figure 2:**
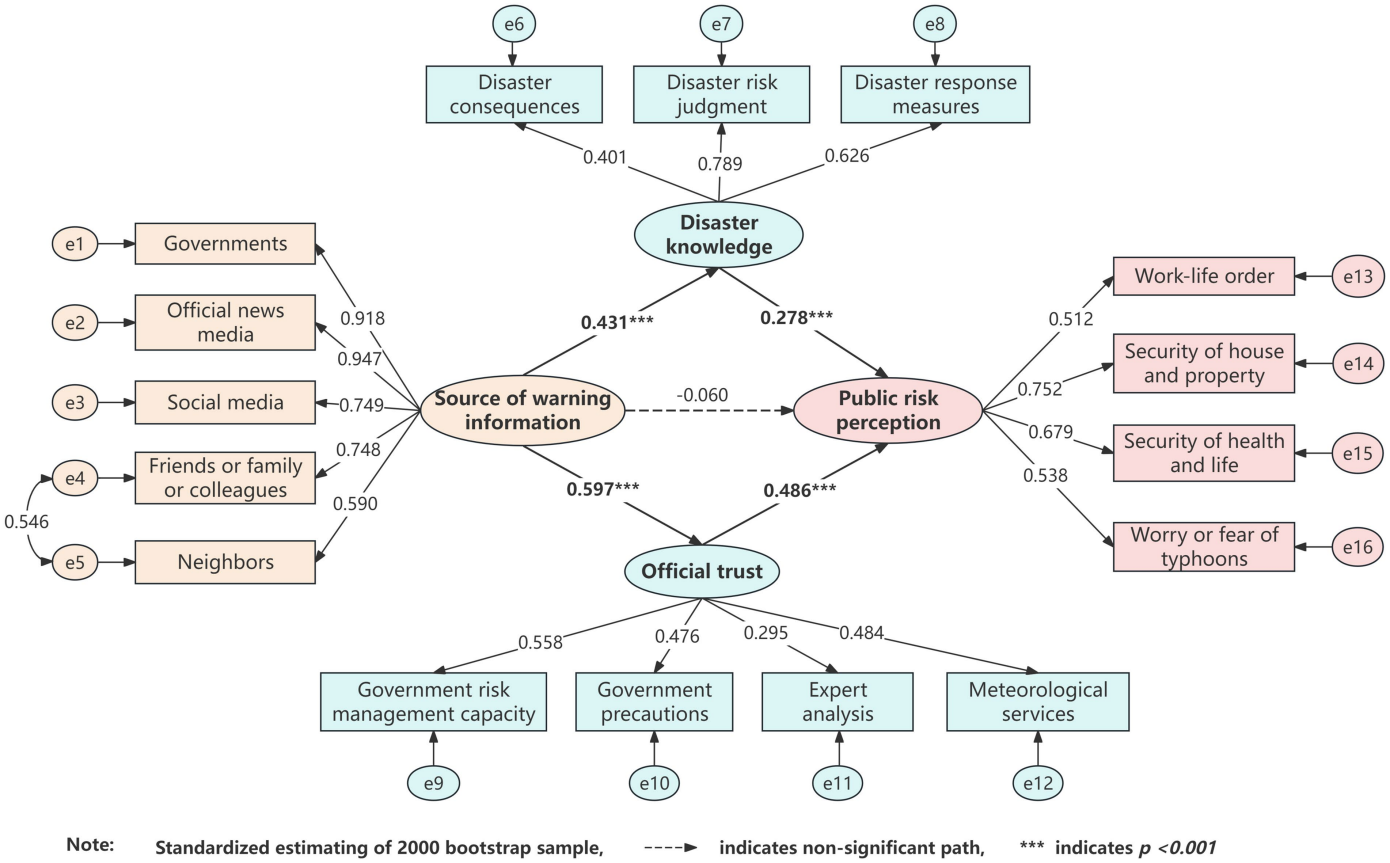
Structural equation modeling of the hypothesized model.

An SEM was used to investigate the association between source of warning information, disaster knowledge, official trust, and risk perception. The SEM fitting index indicated that the hypothesized model fit the data well (*χ*^2^ = 332.211, df = 99, *χ*^2^/df = 3.356 < 5, RMSEA = 0.068 < 0.08, GFI = 0.925, CFI = 0.925, IFI = 0.925, TLI = 0.909). The results showed that disaster knowledge, and official trust are directly associated with risk perception. It is important to note that the source of warning information did not directly associated with risk perception in the SEM model. Figure 2 shows the estimated model with standardized path coefficients.

Table 4 summarizes the unstandardized path coefficients for direct, indirect, and total effects derived from the path analysis. The significance of the mediating effects was assessed using bootstrapping with 2,000 samples, reporting both percentile and bias-corrected 95% confidence intervals (49). The magnitudes of the indirect relationship of source of warning information through disaster knowledge and official trust to risk perception were 0.211, *p* < 0.001. Because zero is not contained in the CI interval, it can be assumed that disaster knowledge and official trust are mediators. The magnitudes of the direct relationship of source of warning information, disaster knowledge, and official trust to risk perception were −0.031 (*p* > 0.05), 0.331 (*p* < 0.01), 0.467 (*p* < 0.01), respectively. The total effects of source of warning information, disaster knowledge, and official trust to risk perception were 0.180(*p* < 0.001), 0.331 (*p* < 0.01), 0.467 (p < 0.01), respectively. The relationship between source of warning information and risk perception was fully mediated by disaster knowledge and official trust.

**Table 4:** Unstandardized, direct, and indirect effects of the hypothesized mode.

Path	Point estimate	*p*	SE	Bootstrapping 2000 times CI			
				Bias-corrected		Percentile	
				Lower	Upper	Lower	Upper
Total effect							
Source of warning information → Public risk perception	0.180	<0.001	0.037	0.115	0.262	0.114	0.259
Disaster knowledge → Public risk perception	0.331	<0.01	0.107	0.166	0.597	0.162	0.586
Official trust → Public risk perception	0.467	<0.01	0.143	0.247	0.823	0.230	0.800
Direct effect							
Source of warning information → Public risk perception	−0.031	>0.05	0.047	−0.141	0.045	−0.138	0.047
Disaster knowledge → Public risk perception	0.331	<0.01	0.107	0.166	0.597	0.162	0.586
Official trust → Public risk perception	0.467	<0.01	0.143	0.247	0.823	0.230	0.800
Indirect effect							
Source of warning information → Public risk perception	0.211	<0.001	0.060	0.119	0.363	0.116	0.349

Unstandardized estimating of 2000 bootstrap sample.

## 4 Discussion

The main purpose of this study was to analyze the influence of the source of warning information, disaster knowledge, and official trust on risk perception following the 2024 super typhoon in Hainan. First, the study found that participants’ some key demographic factors exhibit significant associations with risk perception in typhoon disasters. Moreover, the primary finding indicates that disaster knowledge and official trust are directly and positively associated with risk perception. However, the source of warning information has an indirect correlation with risk perception. The relationship between the source of warning information and risk perception was fully mediated by disaster knowledge and official trust. These findings confirm H1, H2, and H4, but do not support H3. The findings carry theoretical and practical significance. By demonstrating that the OCM theory effectively predicts how warning information sources influence risk perception in typhoons, this study underscores the framework’s utility for explaining risk perception formation. Given the observed individual differences in risk perception and the positive results of disaster knowledge and official trust, targeted educational interventions and authoritative training conferences are recommended to enhance public risk perception to promote proactive disaster preparedness behaviors and strategies.

Research found that higher risk perception was associated with several demographic factors: being female, aged 20–39, having a higher level of education, as well as having a longer residence in Hainan (>5 years), more household members, having multiple children, and prior disaster experience. The public risk perception of disasters, especially at the individual level, has been recognized as a determinant of protective actions, such as evacuation, during disaster warning (10, 22). The survey was conducted after Super Typhoon Yagi made landfall in Hainan, with 91.1% of respondents having experienced typhoon disasters. The physical devastation caused by typhoons likely heightened public risk perception, which aligns with the observed moderately high perception scores. Therefore, residents living Hainan more than 5 years and having disaster experience showed higher risk perception, consistent with the findings of Ma et al. (15). Additionally, females with aged 20–39, more family members and those with multiple children exhibited higher risk perception, a finding that aligns with the work of Billman et al. (10), Ng et al. (14), and Shen et al. (29). This may be due to women generally assuming more family care-giving roles in China, making them more sensitive to safety risks (40). Furthermore, individuals aged 20–39, having children and more family members prompted to consider their family’s safety prior to the arrival of the typhoon (14). Clearly, middle-aged people have heavier family responsibilities, making them particularly aware of potential disruptions and losses (29). Higher education likely enhances the capacity to comprehend complex warning information and objectively assess potential threats, thereby fostering greater risk perception (17). Consequently, government authorities should place greater emphasis on increasing risk perception among men and individuals who have no prior disaster experience, thereby enhancing emergency preparedness motivation.

As source of warning information, disaster knowledge, and official trust toward risk perception are complex constructs that cannot be entirely explained by a single indicator. Based on the OCM theory, the use of SEM and latent constructs provide a more comprehensive measure of these concepts. The results of the correlation analysis and SEM provide additional insights into their complex relationships. First of all, disaster knowledge and official trust are directly and positively associated with risk perception, supporting H1 and H2. This finding aligns with existing literature on knowledge in disaster preparedness, particularly in earthquake-affected areas of China (17). Limited knowledge about disasters leads to lower awareness of risks, resulting in inadequate disaster preparation at the individual level. Some studies have shown that a significant portion of the public, characterized by low-disaster knowledge, often fails to recognize the severity of impending tropical cyclones, accurately predict potential landfall locations, or acquire knowledge about effective response strategies for typhoons (51). Therefore, mastering knowledge about typhoons plays an irreplaceable role in risk perception and risk response (52). The model of disaster education can draw from training experiences in Europe and the United States. The content of education gradually shifts from theoretical instruction to practical exercises, which could enhance residents’ risk perception before improving their capabilities for risk reduction (11).

Trust in government and experts is a crucial factor influencing individuals’ risk perception (32, 53). The result was observed in this study, where official trust had a significant and positive correlation with typhoon risk perception. Contrary to previous research, Bondman and Cisternas reported that residents with high levels of official trust exhibited lower levels of risk perception (18, 19). However, this is not a positive phenomenon. Excessive dependence on the government can diminish individuals’ risk perception and preparedness behavior, undermining their ability to respond proactively to disasters (32). In contrast, during actual disaster response, heightened risk perception and strong official trust may better facilitate disaster preparedness and evacuation actions (54). Trust in authority can influence an individual’s risk perception following experiencing disasters, a process that is quite complex (31, 32). In this research, when faced with a typhoon disaster, individuals with heightened risk perception are more likely to trust the government’s capabilities and its disaster response measures. They are also more inclined to respond positively and comply with the government’s directives and arrangements to ensure their safety. The possible reason is that this study was conducted within 6 months after the disaster. Almost all respondents experienced the typhoon, remained vigilant about the disaster, and were satisfied with the government’s response measures. Therefore, it is suggested that encouraging public participation in disaster management is preferable to relying solely on government decisions.

Critically, the path analysis revealed that the source of warning information had only an indirect correlation with risk perception, which was fully mediated by disaster knowledge and official trust. This unexpected result may stem from the fact that the source of warning information was selected as the only criterion for measuring the information mechanism in this study. However, this study identified disaster knowledge and official trust as significant mediators in the relationship between warning information and risk perception, which empirically validates hypothesis 4. The above results further indicate that, on one hand, the public internalizes the processing of warning information through their disaster knowledge, leading to heightened risk perception and related disaster protection behaviors (22, 28, 54). On the other hand, when receiving warning information, residents with a greater level of official trust are more disaster-aware; they not only take the initiative to engage in preparedness actions but also comply with the government’s disaster response measures (18). A study on natural disasters has shown that individuals who obtain information from the Internet or people around them, along with respondents who perceive a positive attitude toward government authority, exhibit heightened risk perception (55). An Australian study on flood disaster risk communication also found that area-specific warnings were deemed more meaningful due to fragmented perceptions of warning information sources. Thus, enhancing publicity and education about typhoon disaster-related knowledge, utilizing diverse channels for warning information, and strengthening government credibility contribute to elevating risk perception and encouraging disaster protection actions.

### 4.1 Limitations

Despite these significant findings, our study has several limitations that warrant careful consideration. First, the cross-sectional design constrains risk perception assessment to a static timeframe, precluding analysis of its temporal dynamics. Future longitudinal investigations are imperative to elucidate the evolution and persistence of public risk perception. Second, the reliance on an online-recruited convenience sample may impose constraints on the generalization of the results. Non-probability sampling mechanisms amplified non-ignorable sampling error. Willing participants likely differed from non-participants in unmeasured traits (e.g., risk-seeking attitudes, disaster preparedness behavior). Propose targeted, stratified offline recruitment in future research. Third, risk perception was measured using a 5-point Likert scale, which may not capture subtle gradations in perception. Furthermore, as with all self-reported data, responses are subjective and may not perfectly align with actual thoughts or behaviors—a limitation inherent to the method that is difficult to circumvent. However, it is impossible to validate the opinions provided by respondents. Additionally, although the participants we recruited hailed from various regions of the island, ensuring a diverse sample, the fact that the same island with similar traditions and cultures may have had a slight impact on the survey results. Finally, this study concentrated on a predictable typhoon. Other natural hazards (e.g., earthquakes) are unpredictable, so the findings related to typhoon disaster risk perception cannot be generalized to other types of natural hazards.

## 5 Conclusion

This study systematically examines public risk perception after a super typhoon disaster in Hainan Island, China. Analyzing data from an actual typhoon event provides empirical insights into the relationship between risk perception and its constituent components. Residents with lower risk perception were observed among males, and those without children and lacking prior disaster experience in this study. Furthermore, disaster knowledge and official trust completely mediated the association between warning information and risk perception. These findings suggest that in typhoon-prone regions, policymakers may consider exploring diversified warning channels while prioritizing the building of institutional trust. Additionally, targeted disaster risk education could be enhanced for populations with lower risk perception.

## Data Availability

The raw data supporting the conclusions of this article will be made available by the authors, without undue reservation.

## Footnotes

1
www.wjx.cn/
